# Acquired mutations and transcriptional remodeling in long-term estrogen-deprived locoregional breast cancer recurrences

**DOI:** 10.1186/s13058-020-01379-3

**Published:** 2021-01-06

**Authors:** Nolan Priedigkeit, Kai Ding, William Horne, Jay K. Kolls, Tian Du, Peter C. Lucas, Jens-Uwe Blohmer, Carsten Denkert, Anna Machleidt, Barbara Ingold-Heppner, Steffi Oesterreich, Adrian V. Lee

**Affiliations:** 1 Department of Medicine, Brigham and Women’s Hospital/Harvard Medical School, Boston, MA USA; 2 Women’s Cancer Research Center, UPMC Hillman Cancer Center, Pittsburgh, PA USA; 3 Department of Pharmacology and Chemical Biology, University of Pittsburgh, Pittsburgh, PA USA; 4 Magee-Women’s Research Institute, Magee-Women’s Research Hospital of University of Pittsburgh Medical Center, Pittsburgh, PA USA; 5 Richard King Mellon Foundation Institute for Pediatric Research, UPMC Children’s Hospital of Pittsburgh, Pittsburgh, PA USA; 6 Department of Pathology, University of Pittsburgh School of Medicine, Pittsburgh, PA USA; 7 Institute of Pathology and Department of Gynecology, Charité University Hospital, Berlin, Germany; 8 Institute of Pathology, Philipps-University Marburg and University Hospital Marburg (UKGM), Marburg, Germany; 9 Institute of Pathology, DRK Kliniken Berlin, Berlin, Germany; 10 Department of Human Genetics, University of Pittsburgh, Pittsburgh, PA USA; 11 Magee-Women’s Research Institute, 204 Craft Avenue (Room A412), Pittsburgh, PA 15213 USA

**Keywords:** Breast cancer, Estrogen receptor, Locoregional recurrence, Endocrine therapy, Therapy resistance, RNA-seq, DNA-seq, Copy number alterations, FFPE, Tumor profiling, Exome capture, Targeted sequencing, Cancer genomics, *ESR1*, *NTRK*, *ARID1A*

## Abstract

**Background:**

Endocrine therapy resistance is a hallmark of advanced estrogen receptor (ER)-positive breast cancer. In this study, we aimed to determine acquired genomic changes in endocrine-resistant disease.

**Methods:**

We performed DNA/RNA hybrid-capture sequencing on 12 locoregional recurrences after long-term estrogen deprivation and identified acquired genomic changes versus each tumor’s matched primary.

**Results:**

Despite being up to 7 years removed from the primary lesion, most recurrences harbored similar intrinsic transcriptional and copy number profiles. Only two genes, *AKAP9* and *KMT2C*, were found to have single nucleotide variant (SNV) enrichments in more than one recurrence. Enriched mutations in single cases included SNVs within transcriptional regulators such as *ARID1A*, *TP53*, *FOXO1*, *BRD1*, *NCOA1*, and *NCOR2* with one local recurrence gaining three *PIK3CA* mutations. In contrast to DNA-level changes, we discovered recurrent outlier mRNA expression alterations were common—including outlier gains in *TP63* (*n* = 5 cases [42%]), *NTRK3* (*n* = 5 [42%]), *NTRK2* (*n* = 4 [33%]), *PAX3* (*n* = 4 [33%]), *FGFR4* (*n* = 3 [25%]), and *TERT* (*n* = 3 [25%])*.* Recurrent losses involved *ESR1* (*n* = 5 [42%]), *RELN* (*n* = 5 [42%]), *SFRP4* (*n* = 4 [33%]), and *FOSB* (*n* = 4 [33%]). *ESR1-*depleted recurrences harbored shared transcriptional remodeling events including upregulation of *PROM1* and other basal cancer markers.

**Conclusions:**

Taken together, this study defines acquired genomic changes in long-term, estrogen-deprived disease; highlights the importance of longitudinal RNA profiling; and identifies a common *ESR1-*depleted endocrine-resistant breast cancer subtype with basal-like transcriptional reprogramming.

## Background

Hormone receptor-positive breast cancer has served as a prototype for targeted therapy due to the well-established efficacy of estrogen deprivation. Largely because of these approaches, breast cancers are somewhat unique in that recurrences can occur years, sometimes decades following the primary diagnosis [1–4]. Given that the majority of patients receive long-term maintenance regimens of either a selective estrogen receptor modulator (SERM) or aromatase inhibitor (AI), recurrent breast cancers are often classified as estrogen-independent given their ability to thrive in an estrogen-deprived environment. Identifying the biological mediators that allow breast cancer cells to bypass their dependence on estrogen is a crucial step in understanding advanced breast cancer biology and defining novel therapeutic targets.

Defining these molecular processes in patient samples, however, has been challenging because of the logistics in obtaining well-characterized, longitudinally collected biospecimens. Nevertheless, shared features of more advanced breast cancers have emerged, such as relapsed tumors losing expression of ER and over 20% of metastatic ER-positive breast cancers acquiring mutations in *ESR1* that confer ligand-independent signaling [5–7]. Other largely accepted mechanisms of estrogen-independence are bypass activations of mitogenic pathways such as MAPK and PI3K through initiating FGFR, EGFR, and IGF signaling and exploitation of the Rb-CDK-E2F axis [8–12]. Less well validated, more recently discovered mechanisms include *ESR1* fusions and amplifications [13, 14].

Recent studies analyzing multiple, longitudinally collected, pre- and post-treatment samples have shown clonal evolution and selection in the context of targeted therapies [15–18]. Similar work analyzing hormone receptor-positive breast cancers have largely been restricted to short-term pre-/post-neoadjuvant therapy analyses [19–22]. One of the most comprehensive genomic studies of this type was a multi-platform effort that characterized the clonal architecture of tumors after 4 months of AI therapy [23]. Although drastic clonal remodeling was observed at the DNA level, few recurrent resistance mechanisms were appreciated. A more recent, large-scale study showed activating *ERBB2* mutations, MAPK activation, and NF1 loss as mechanisms possibly driving endocrine resistance—with some of these alterations being confirmed in subsequent studies [24–27]. The majority of this work has notably been performed on metastatic tissues—whether or not some of these changes occur locally as a result of estrogen independence before distant spread is unknown.

Thus, to better define both DNA and transcriptional changes that occur in long-term estrogen-independent tumors, we undertook a targeted analysis of DNA/RNA alterations in ~ 1400 cancer genes in 12 paired primary and locoregional recurrences from patients with ER-positive breast cancers that were documented as being treated with estrogen-depleting therapy. The median time to recurrence was 3.7 years, with the longest time to recurrence being over 7 years.

## Methods

### Patient samples, tissue processing, and nucleic acid extraction

Institutional Review Board approval from both participating institutions (University of Pittsburgh IRB# PRO15050502, The Charité IRB Office) was obtained prior to initiating the study. Inclusion criteria for this study were (1) patients harbored patient-matched formalin-fixed paraffin-embedded (FFPE) tissue from primary breast cancers and local recurrences, (2) biospecimens contained macrodissectable regions with sufficient tumor cellularity, and (3) disease was treated continuously with a form of estrogen-depleting therapy prior to the recurrence. Biospecimens were reviewed by a trained molecular pathologist to confirm pathology, to quantify tumor cellularity, and to highlight regions of relatively high tumor cellularity for macrodissection. If a slide region harbored sufficient, microscopically verifiable adjacent normal cells, this region was also dissected and processed for downstream analyses. Between four to ten (depending on tumor size) 10-μm FFPE sections immediately adjacent to the H&E-analyzed section were pooled and underwent dual DNA/RNA extraction using Qiagen’s AllPrep kit. Nucleic acids were quantified fluorometrically with a Qubit 2.0 Fluorometer and quality assessed with an Agilent 4200 TapeStation Instrument prior to sequencing.

### RNA and DNA sequencing

RNA-seq library preparation was performed for all 12 cases using approximately 100 ng of RNA and Illumina’s *TruSight RNA Pan-Cancer* (1385 targets) protocol. DNA-seq library preparation was performed for 10 (6 with associated normal tissue, 2 cases were excluded based on limiting DNA content) cases using no less than 30 ng of DNA and Illumina’s *TruSeq Exome* protocol with *TruSight RNA Pan-Cancer* probes for hybridization-based capture. Indexed, pooled libraries were then sequenced on Medium Output flow cells using an Illumina NextSeq 500 system (paired-end reads, 2 × 75 bp). A target of 5–10 million reads per sample was used to plan indexing and sequencing runs for RNA sequencing, and a target of 10–15 million reads was used for DNA sequencing. Additional RNA-seq data derived from luminal metastatic breast cancers were obtained from the MET500 cohort (*n* = 47) [28] and our own University of Pittsburgh’s Women’s Cancer Research Institute (WCRC) cohort (*n* = 89) [29–31]. All RNA-sequencing FASTQ files were quantified with k-mer-based lightweight-alignment (*Salmon*, quasi-mapping mode, 31-kmer index using GRCh38 Ensembl v82 transcript annotations, seqBias and gcBias corrections) [32]. *tumorMatch* was used to validate sequencing pairs were patient-matched as done previously [30].

### RNA-sequencing quantification and DNA-sequencing alignment

RNA-seq read counts and mapping percentages were calculated (Data Supplement S1) and transcript abundance estimates were collapsed to gene level with tximport [33]. Log2-transformed TMM-normalized CPM (log2normCPM) values were implemented for subsequent analyses [34, 35]. DNA-seq reads were aligned with *bwa –mem* (v.0.7.13) to an hg19 reference, sorted with *samtools* (v1.3), duplicates marked and removed with *picardtools* (v1.140), and local realignment performed with *GATK* (v3.4-46) [36–38]. Average coverage depth for the processed bam file was calculated using *GATK’s DiagnoseTargets* and the Illumina *Pan-Can* bed file (Data Supplement S2). Metrics for average coverage values across all target intervals were plotted with *ggplot2.* Publicly available RNA sequencing or microarray data of ER-positive breast cancer cell lines in the absence or presence of E2 were collected from GEO, including MCF7_1 (GSE89888), MCF7_2 (GSE51403), MCF7_3 (GSE78286), MCF7_4 (GSE94493), T47D_1 (GSE89888), T47D_2 (GSE3834), T47D_3 (GSE3834), T47D_4 (GSE108304), ZR75-1 (GSE61368), BT474 (GSE3834), MM134 (GSE50695), and SUM44 (GSE50695). Cells were deprived in E2-free media, stimulated with vehicle (Veh) or 1 nM/10 nM E2 for 16 or 24 h, then processed from gene expression profiling. *KLK7* and *PROM1* Log2 (TPM+1) expression value were compared between Veh and E2-treated group of each cell line.

### DNA-seq recurrence-enriched variant determination

To determine enriched variants in recurrences versus patient-matched primary tumors, *VarScan2* was implemented [39]. More specifically, primary and recurrent *samtools* mpileup files derived from processed bam files were input into *VarScan2* using *somatic* mode, with somatic *p* values representing the significance of a particular variant being acquired or enriched in the recurrence [SS = 1 or SS = 2]. Tumor purity estimates, as assessed by a molecular pathologist, were included in *VarScan2* to correct contaminating normal cell influence on allele frequencies. The minimum coverage for a variant to be considered was 40X, with a minimum allele frequency (AF) of 0.05 in either the primary or recurrence and a minimum of 5 reads supporting the variant. Germline variants were determined for cases containing a matched normal (ERLR_01, ERLR_02, ERLR_07, ERLR_08, ERLR_12, and ERLR_15) using *VarScan2*’s *germline* mode with the same parameters. VCF output files were then imported into R using the *VariantAnnotation* package [40]. If a normal sample was available for the case, all germline variants (AF > 0.30) were excluded from subsequent analyses. Additionally, to limit technical artifacts especially considering specimens were formalin-fixed paraffin-embedded [41], a “blacklist” of variants was created including those called in at least 3 of the normal samples. Germline and blacklist-removed variants were then annotated with *Annovar* [42]. Lastly, to call recurrence-enriched, potentially pathogenic variants, the following inclusion criteria were enacted: (1) VarScan2 somatic *p* value < 0.05, (2) > 2-fold gain in allele frequency in the recurrence versus the primary, (3) minimum AF of 0.10 in the recurrence, (4) non-silent, and (5) an ExAC AF < 0.01 considering some samples were without a paired normal [43]. These non-silent, enriched, potentially pathogenic variants were then plotted using the *OncoPrint* function in *ComplexHeatmaps* [44]. A Pearson R correlation was calculated between the frequency of enriched variants and disease-free survival. *PIK3CA* mutations were visualized with *IGV* (2.3.60) [45] and variant allele frequencies were derived from *VarScan2*.

### RNA-seq variant determination

RNA-seq reads covering mutation sites called from DNA-seq of the corresponding sample were extracted from bam file and counted. Variants with at least 2 supporting reads containing the altered allele and with AF greater than 0.05 in either primary or recurrence were considered.

### Copy number alterations

To estimate copy number ratios, *CNVkit* was implemented on processed bam files using default settings and the *-drop-low-coverage* option [46]. A pool of bam files from adjacent normal tissue, sequenced in the same manner, was used as a reference. Probe- and segment-level copy number estimates were finalized with *CNVkit*’s *call* function, which utilizes circular binary segmentation [47]. To adjust for tumor purity and normal contamination, the *–m clonal* option was used with tumor purities from pathologic evaluations. Copy number ratios were then plotted with the *heatmap* function, and copy number values were assessed and plotted with *ggplot2*. Gene-level copy number estimates represent the mean copy number call across all probe targets.

### Differential gene expression, clustering, and outlier gains and losses

Hierarchical clustering was performed using the heatmap.3 function (https://raw.githubusercontent.com/obigriffith/biostar-tutorials/master/Heatmaps/heatmap.3.R) in R on log2normCPM values of the top 10% most variable genes (defined by IQR) with 1 minus Pearson correlations as distance measurements and the “average” agglomeration method. Differential expression between primary and recurrent tumors was analyzed with *limma*. Raw counts were input into the *voom* function and quantile normalized prior to fitting the linear model and performing the empirical Bayes method for differential expression [48, 49]. The linear model was fitted with a design that accounts for the paired nature of the cohort (model = ~Patient+Tissue [primary or recurrence]). Outlier expression gains and losses were determined for each patient by discretely categorizing genes into one of 5 categories. If log2FC values (i.e., recurrence log2normCPM – primary log2normCPM) for a given gene were less than Q1 – (1.5 × IQR) or Q1 – (3 × IQR), using case-specific log2FC values for all genes as the distribution, that gene was deemed an “Outlier Loss” or “Extreme Loss” respectively. If log2FC values calculated were greater than Q3 + (1.5 × IQR) or Q3 + (3 × IQR), it was deemed an “Outlier Gain” or “Extreme Gain” respectively. All other genes with intermediate fold changes were classified as “Stable.” To determine subtype expression of *KLK7*, *PROM1*, and *NDRG1*, normalized microarray expression data along with PAM50 calls was obtained from the Molecular Taxonomy of Breast Cancer International Consortium (METABRIC) through Synapse (https://www.synapse.org/, Synapse ID: syn1688369), following IRB approval for data access from the University of Pittsburgh [50]. Overlap with genes in long-term estrogen-deprived, ER-positive breast cancer lines (HCC1428, MCF7, T47D, ZR75.1) was performed by running a separate differential expression analysis (LTED vs. parental lines) on microarray data with *limma* [49, 51]*.* Dysregulated gene overlap was designated if the nominal *p* value and FDR-adjusted *p* value were both < 0.05 in the local recurrence and LTED differential expression analysis, respectively. Binary dichotomization of METABRIC samples using *NDRG1* expression (> 50th percentile, < 50th percentile) and log-rank testing were used to assess significant differences in disease-specific survival (DSS) and then Kaplan-Meier curves were plotted with *survminer* [52, 53].

## Results

### Expression and copy number changes in local recurrences

**Fig. 1:**
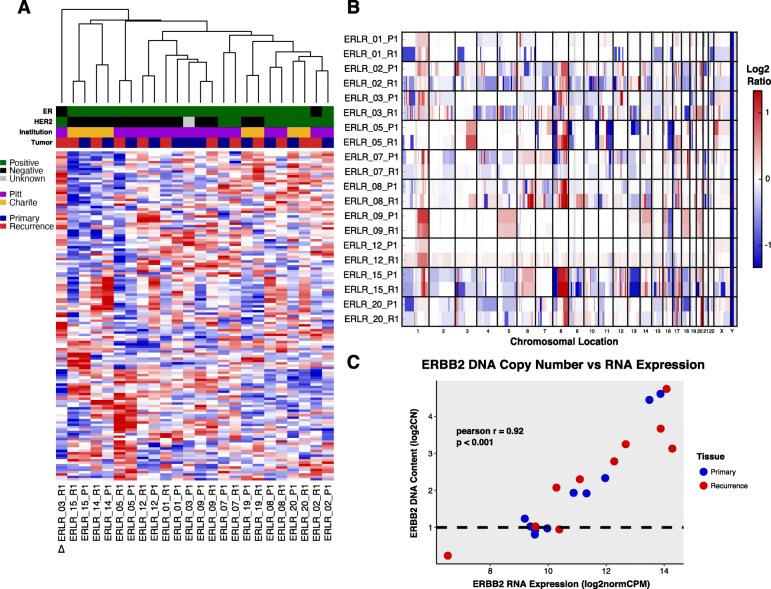
Transcriptional and CNA profiles of ER-positive local recurrences. **a** Unsupervised hierarchical clustering and heatmap (red = high relative expression, blue = low relative expression) on normalized gene expression values from patient-matched pairs (P1 = primary, R1 = recurrence). Clinical ER and HER2 status (black = negative, green = positive, gray = unknown), tissue source site (purple = Pitt, yellow = Charite), and tumor type (blue = primary, red = recurrence) are indicated. Delta symbol shows distinct clustering of ERLR_03_R1 away from its matched primary, ERLR_03_P1. **b** Heatmap of copy number ratios from patient-matched pairs. Redder regions indicate regions of copy number gain and bluer regions indicate regions of loss. **c** Correlation between ERBB2 DNA copy number calls and normalized expression values

Dual hybrid-capture DNA/RNA sequencing was performed for 1385 cancer genes on 12 paired primary tumors and local recurrences from patients with ER-positive breast cancers that underwent continuous endocrine therapy (Table 1). RNA-seq data (Supplementary Table 1) underwent unsupervised hierarchical clustering of normalized RNA expression values which showed most patient-matched pairs clustered transcriptionally with their matched primary—regardless of the length of disease-free survival (Fig. 1a). Unlike a previous transcriptome-wide analysis of primary breast cancers and matched bone metastases [30], there was no significant correlation in pair transcriptional similarity and time to recurrence—although a trend towards negative correlation was observed (Pearson *R* = − 0.37, *p* value = 0.236). Only a single recurrence showed marked transcriptional deviation from its matched primary (ERLR_03_R1); whereby it lost ER positivity and gained HER2 positivity clinically. Copy number alterations (CNAs) between primary and recurrences were analyzed in the targeted capture regions for 10 cases (Supplementary Figure 1 and Supplementary Figure 2). Similar to expression, CNAs were largely consistent among the recurrences when compared to their matched primary (Fig. 1b). Two exceptions were recurrences from cases ERLR_01 and ERLR_03, which showed distinct copy number profiles from the matched primary tumors with poor correlation between primary and recurrence CNA values versus all other cases (Supplementary Figure 3). Notably, unlike case ERLR_03, ERLR_01 interestingly retained a similar expression profile despite a distinct CNA profile. An analysis of shared variants validated both DNA and RNA extracts originated from the same patient (Supplementary Figure 4), excluding the possibility of sample mixup. *ERBB2* copy number values correlated well with RNA expression (Pearson *R* 0.92), as did other amplified genes including *CDK12* and *CCND1* (Fig. 1c, Data Supplement S3).

**Table 1:** Abridged clinicopathological features of patient-matched primary and local recurrence tumor cohort

Case	Age Dx	Hist	Stage	ER Prim	PR Prim	HER2 Prim	Endo Tx	HER2 Tx	Radio Tx	Chemo Tx	DFS	SPLR	Vital status	OS
**ERLR_01**	36	IDC/ILC Mixed	I	Pos	Pos	Neg	Yes	No	Yes	Yes	86	132	Alive	218
**ERLR_02**	54	IDC	IIA	Pos	Neg	Pos	Yes	No	Yes	Yes	61	141	Alive	203
**ERLR_03**	74	IDC	I	Pos	Pos	NA	Yes	No	Yes	No	76	128	Dead	204
**ERLR_05**	54	IDC	IIA	Pos	Pos	Neg	Yes	No	Yes	Yes	69	85	Dead	155
**ERLR_07**	58	IDC	I	Pos	Pos	Pos	Yes	No	Yes	No	19	179	Alive	199
**ERLR_08**	52	IDC	IA	Pos	Pos	Pos	Yes	Yes	Yes	Yes	37	38	Alive	75
**ERLR_09**	51	IDC	IA	Pos	Pos	Neg	Yes	No	Yes	No	25	46	Alive	71
**ERLR_12**	47	IMC	IIA	Pos	Pos	Neg	Yes	No	No	No	26	34	Alive	61
**ERLR_14**	50	IDC	IA	Pos	Pos	Neg	Yes	No	NA	No	3	26	Alive	29
**ERLR_15**	65	IDC	IIIC	Pos	Pos	Neg	Yes	No	Yes	No	10	27	Alive	38
**ERLR_19**	49	IDC w/ lobular features	IIA	Pos	Pos	Neg	Yes	No	No	No	52	8	Alive	61
**ERLR_20**	42	IDC	IIIA	Pos	Pos	Pos	Yes	Yes	Yes	Yes	59	44	Dead	104

*Abbreviations*: *Dx* diagnosis, *Hist* histology, *ER* estrogen receptor, *PR* progesterone receptor, *HER2* human epidermal growth factor 2, *Endo* endocrine, *Tx* therapy, *DFS* disease-free survival, *SPLR* survival post-local recurrence, *OS* overall survival, *IDC* invasive ductal carcinoma, *ILC* invasive lobular carcinoma, *IMC* invasive mucinous carcinoma

### SNV enrichments and differentially expressed genes

**Fig. 2:**
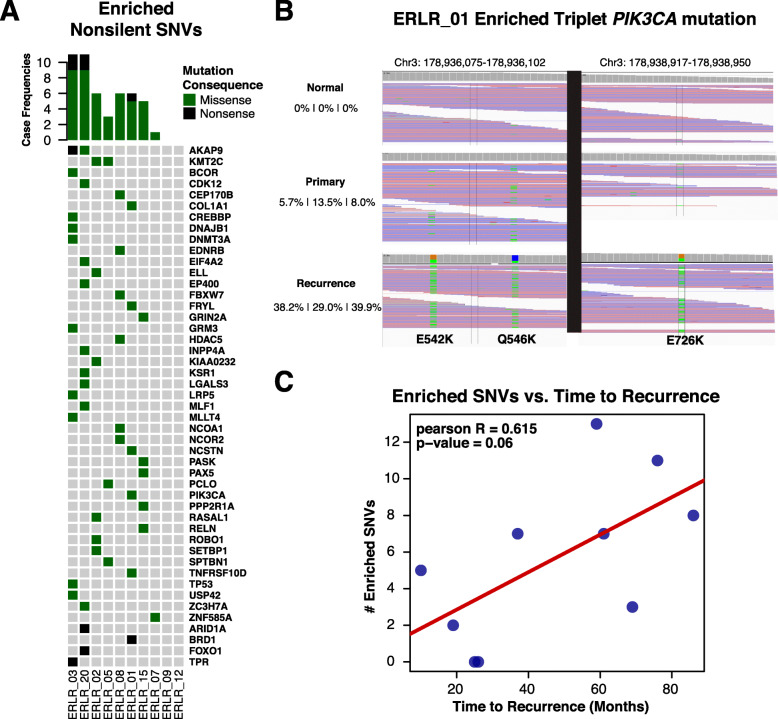
SNV enrichments in ER-positive local recurrences. **a** OncoPrint of non-silent, enriched single nucleotide variants in patient-matched cases. Missense variants are indicated with a green box and nonsense variants with black. **b** Triplet mutation enrichment of PIK3CA mutations in case ERLR_01. Collapsed IGV alignments are shown, along with allele frequencies, for the normal, primary, and recurrence. **c** Frequency of enriched, non-silent single nucleotide variants versus time to recurrence along with Pearson *R* and calculated *p* value

A total of 406 distinct, presumed-somatic nonsynonymous mutations were detected in either a primary or recurrence at an AF > 5% among the 10 DNA-sequenced cases (Data Supplement S4). To assess if there are shared DNA mutations acquired in recurrences, an analysis of enriched single nucleotide variants (SNVs) was performed which showed 56 statistically enriched SNVs in local recurrences versus matched primary tumors (Fig. 2a, Data Supplement S5). SNVs in two genes were found to be enriched in more than one case (*n* = 2 [20%]), *AKAP9* (R3320W, S319*) and *KMT2C* (T1969I, Y366N, R894Q)*.* The recurrent mutations did not exhibit features suggesting functional selection, such as being within a conserved functional domain or within a COSMIC [54] hotspot region, making it difficult to assess if these are pathogenic. Other case-specific, *n*-of-one enriched mutations included nonsense mutations in *ARID1A* (Q1424*, case ERLR_20, primary AF 0.5%, recurrence AF 16.5%) and *BRD1* (Q467*, case ERLR_01, primary AF 0.93, recurrence AF 57.88%), an acquired *TP53* mutation (S241C, case ERLR_03, primary AF 0.0%, recurrence AF 53.4%), and an enriched *NCOR2* mutation (A4942C, case ERLR_08, primary AF 4.4%, recurrence AF 19.4%). In case ERLR_01, an enrichment of a suite of three somatic mutations in *PIK3CA* was observed (E542K, Q546K, E726K) in the recurrence (Fig. 2b). Notably, the number of enriched, non-silent SNVs ranged from 0 to 13 and was positively correlated with clinical time to recurrence (Fig. 2c). No acquired *ESR1* mutations were observed. These mutations were examined in the corresponding RNA-seq data to determine if they are expressed. Out of 633 total mutations—considering some of the 406 distinct mutations were present in both matched tumors—315 were detected in RNA with at least 2 supporting reads of the altered allele and an AF ≥ 5%. Allele frequencies called from DNA-seq and RNA-seq data correlated well (Supplementary Figure 5, Pearson *R* = 0.609, *p* value = 2.57e−33). Noteworthy, out of the 56 enriched SNVs in recurrence at the DNA level, 31 distinct mutations can be detected with confidence at the RNA level (Data Supplement S6)—including *AKAP9*, *KMT2C*, *ARID1A*, *BRD1*, and *TP53* as discussed above.

A differential expression analysis, comparing all primary tumors versus all local recurrences, yielded no genes passing an FDR-corrected *p* value of less than 0.05—which is perhaps expected given the heterogeneity of clinical specimens and the limited number of cases (Data Supplement S7). Nonetheless, 71 genes with an average, *voom* normalized expression value of 2 or greater, a nominal *p* value of less than 0.05, and a log2 fold-change greater than ± 0.5 were identified (Table 2). Some of these genes, including the upregulation of *EPOR*, *NDRG1*, *IDH2*, *CEBPA*, and *PTPA* and downregulation of *ESR1*, *IGF1R*, *NFKB1*, and *RUNX2*, are also differentially expressed in long-term estrogen-deprived ER-positive cell lines (Supplementary Figure 6) [55].

**Table 2:** Differentially expressed genes in ESR1-depleted recurrences

Gene	Log2FC	***voom*** average expression	Nominal ***p*** value	FDR-adjusted ***p*** value
***PAPPA***	1.395	6.416	0.001	0.293
***KLK7***	5.422	1.158	0.001	0.293
***PROM1***	3.931	5.005	0.002	0.588
***RASGRF1***	2.307	4.106	0.002	0.588
***DKK1***	2.732	0.473	0.004	0.614
***EPHB6***	1.641	3.819	0.005	0.614
***ABCC3***	1.637	8.010	0.006	0.614
***FGFR4***	1.515	5.267	0.010	0.695
***FBN2***	1.010	5.326	0.010	0.695
***TENM1***	1.326	4.709	0.012	0.705
***COL9A3***	2.034	2.249	0.014	0.705
***NDRG1***	1.218	8.945	0.014	0.705
***TP63***	2.135	4.441	0.018	0.768
***SCN8A***	1.290	5.881	0.019	0.768
***KIT***	1.289	6.020	0.020	0.768
***TCL6***	2.228	−0.254	0.022	0.790
***WNT11***	1.585	1.256	0.024	0.823
***SOCS1***	1.534	0.387	0.033	0.911
***HOXD11***	2.755	−1.369	0.034	0.911
***PLAG1***	1.275	4.576	0.036	0.911
***DTX4***	1.185	5.711	0.036	0.911
***FLNC***	1.588	6.787	0.037	0.911
***ALDOC***	1.494	5.224	0.039	0.911
***ACSBG1***	1.843	0.601	0.042	0.915
***SYP***	1.348	0.862	0.045	0.915
***ESR1***	− 3.952	9.492	0.000	0.146
***ATP8A2***	− 2.599	4.510	0.003	0.588
***ELOVL2***	− 2.090	2.413	0.006	0.614
***RABEP1***	− 1.009	10.352	0.012	0.705
***EYA1***	− 1.494	2.203	0.013	0.705
***IGF1R***	− 1.149	9.083	0.016	0.747
***CAMK2A***	− 1.391	2.742	0.016	0.747
***RERG***	− 1.413	6.562	0.018	0.768
***BCL2***	− 1.055	6.619	0.020	0.768
***FGF14***	− 1.393	2.430	0.023	0.790
***RASGRP1***	− 1.044	6.799	0.027	0.857
***BHLHE22***	− 1.822	0.822	0.035	0.911
***ZNF703***	− 1.811	4.865	0.038	0.911
***MYB***	− 1.179	8.857	0.045	0.915

### Outlier expression gains and losses

**Fig. 3:**
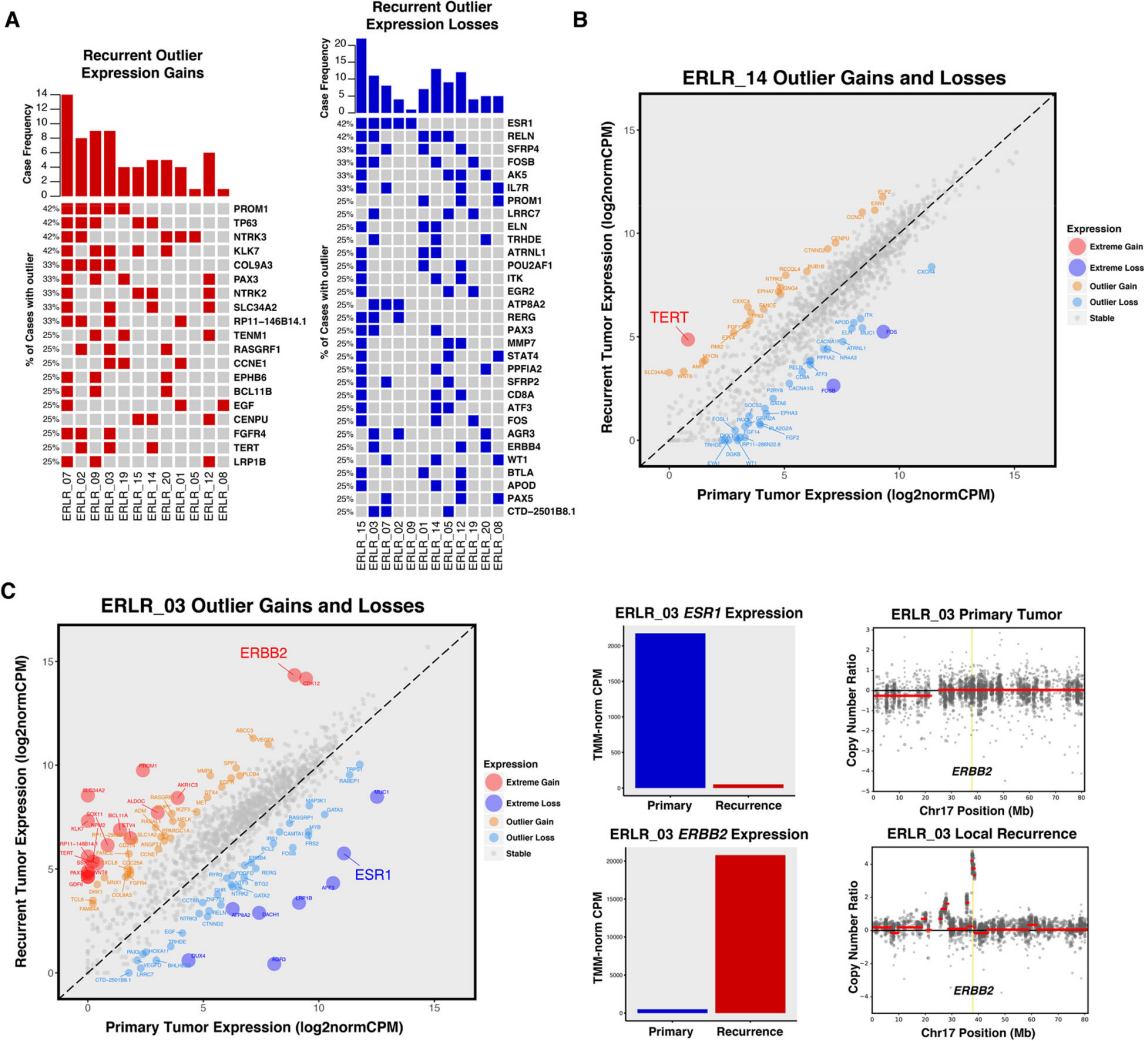
Outlier expression gains and losses in ER-positive local recurrences. **a** OncoPrint of outlier expression gains (red) and outlier expression losses (blue) in ER-positive local recurrences. Genes are sorted by frequency of outlier changes across pairs. **b** Extreme expression gain of TERT in case ERLR_14; 2 other cases showed similar TERT enrichments in recurrent tumors. **c** Extreme expression gain and loss of ERBB2 and ESR1, respectively. TMM-normalized CPM values of primary (blue) and recurrent (red) tumor. ERBB2 expression gain is driven by recurrence-specific DNA-level amplification of ERBB2 locus

To further explore major expression changes that may be driving recurrence and therapy resistance, an outlier expression analysis was performed using gene-level fold-change values of each patient-matched case (Data Supplement S8). Unlike non-silent SNVs, recurrent transcriptional gains and losses were common (Fig. 3a). These included gains and losses in shared pathway members, notably *NTRKs* and *SFRPs*, respectively; targetable upregulation of growth factor pathway mediators such as *FGFR4* and *EGF*; and outlier gains in the CDK regulator *CCNE1*. Three of 12 cases also shared outlier expression gains in *TERT*, with case ERLR_14 harboring a particularly extreme enrichment from near undetectable levels in the primary tumor (Fig. 3b). Case ERLR_03’s recurrence, which was most dissimilar to its patient-matched pair transcriptionally, showed extreme loss and gain of *ESR1* and *ERBB2*, respectively. CNA analysis confirmed recurrence-specific *ERBB2* amplification and is consistent with previous studies of endocrine therapy-treated breast cancers selecting for HER2 signaling in more advanced tumors. The most recurrent outlier loss involved *ESR1*.

### ESR1 depleted recurrences

**Fig. 4:**
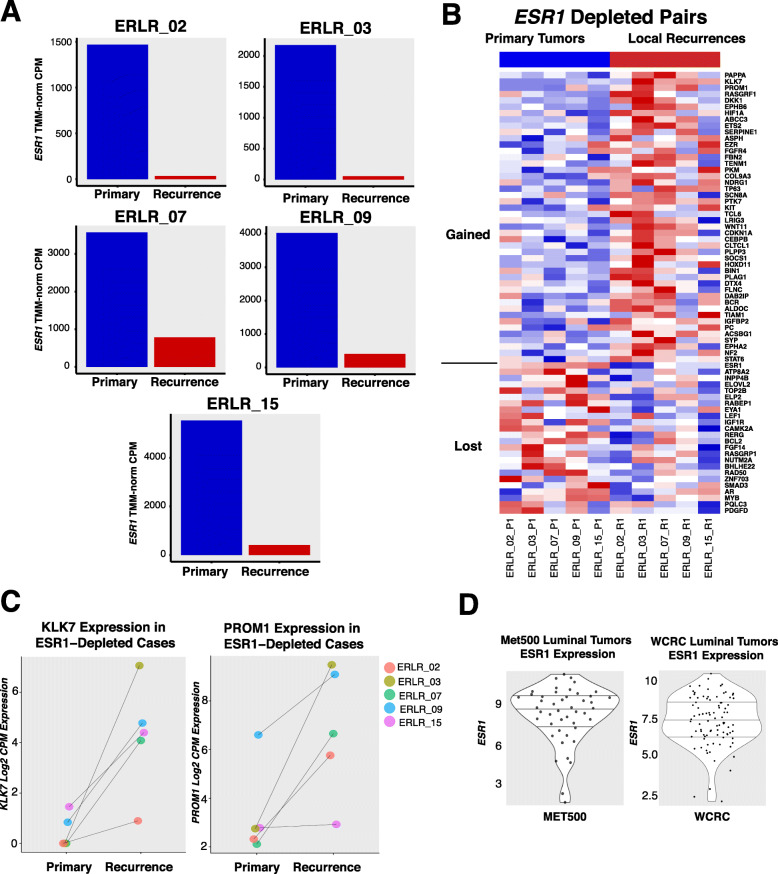
ESR1-depleted recurrences. **a** TMM-normalized expression of patient-matched local recurrences; primary tumor expression in blue, recurrent tumor expression in red. **b** Heatmap of differentially expressed genes (nominal *p* value < 0.05, red = high relative expression, blue = low relative expression) in ESR1-depleted recurrences versus matched primary tumors. Genes are sorted by *p* value and segregated by log2 fold-change values; log2 fold change > 0 on top, log2 fold change < 0 on bottom. **c** Ladder plots showing log2normCPM expression values for both KLK7 and PROM1, two of the most significantly upregulated genes in local recurrences with the largest average log2 fold changes. **d** ESR1 expression in metastatic, endocrine-resistant luminal tumors—MET500 (*n* = 47) and WCRC (*n* = 89) cohorts

Five cases showed outlier expression losses of *ESR1* (Fig. 4a). Despite estrogen receptor being the driver of ER-positive breast cancer and a major regulator of transcription, counterintuitively, 4 of 5 of the recurrences which lost *ESR1* expression generally retained the expression profile of their patient-matched primary (Fig. 1a). Importantly, many of these cases also harbored very similar CNA profiles (Fig. 1b), implying the recurrences were derived from a continuous clonal lineage as opposed to being completely distinct breast cancers. Thus, to explore the transcriptional consequences of acquired *ESR1* loss in ER-positive disease and identify potential bypass mechanisms driving ER*−* independence, a differential expression analysis was performed on the subset of pairs with outlier *ESR1* expression losses. This analysis revealed several recurrently dysregulated genes in *ESR1*-depleted recurrences (Fig. 4b, Data Supplement S9). Two standout genes, *KLK7* and *PROM1*, showed the highest degree of fold change with a log2 fold-change increase of 5.4 and 3.9 respectively—with some tumors exhibiting changes from near undetectable levels to high expression (Fig. 4c). These two genes are more commonly expressed in basal cancers, with *PROM1* being a cancer lineage stem cell marker and perhaps negatively regulated by E2 stimulation (Supplementary Figure 7) [56]. Other genes with significant log2 fold changes > 1 included drug targets such as *FGFR4*, *KIT*, *IGF1R*, and *BCL-2* (Table 2). *NDRG1*, a particularly compelling candidate since it also showed upregulation in LTED breast cancer models, was further interrogated using METABRIC data. Like *PROM1* and *KLK7*, *NDRG1* is most highly expressed in basal breast cancers; yet, when expressed in ER-positive primary tumors, *NDRG1* confers significantly worse disease-specific survival outcomes (Supplementary Figure 8). Notably, none of the top differentially regulated genes in ESR1-depleted recurrences showed statistically significant changes in expression in non-ESR1-depleted recurrences (Supplementary Figure 9). To determine the prevalence of ESR1-depleted in other endocrine-resistant cohorts, we analyzed RNA-seq expression data from the MET500 and our own WCRC cohorts. Although we found most metastatic tumors with PAM50 luminal classifications had high expression of *ESR1* as expected, there was a subset of metastatic luminal tumors which harbored very low levels of *ESR1* expression (Fig. 4d)—suggesting *ESR1* depletion in endocrine-resistant luminal tumors is not restricted to local recurrences and may also be a feature of more advanced disease.

## Discussion

In this study, a targeted RNA/DNA analysis of approximately 1400 cancer genes in ER-positive primary breast cancers and matched long-term, endocrine therapy-treated local recurrences was performed. We found general conservation of transcriptional and copy number profiles among the majority of samples—suggesting that even after 7 years of dormancy and the selective pressures of therapies, locally recurrent breast cancers generally retain their intrinsic molecular features. An analysis of recurrence-enriched SNVs revealed limited recurrent mutation events, yet notable “n-of-one” mutation selection was observed—such as case ERLR_01 which showed three distinct, recurrence-enriched *PIK3CA* mutations. The most striking changes in long-term estrogen-deprived tumors, however, were highly recurrent (up to 42%), outlier expression changes. An analysis of tumors with the most recurrent outlier loss, *ESR1*, revealed concurrent upregulation of genes typically expressed in basal breast cancers, such as *PROM1*, *KLK7*, and *NDRG1*, suggesting a selection of a more basal-like phenotype in endocrine-resistant disease. Our data showing similar CNA profiles argue against the outgrowth of a distinct ER-negative subclone but instead suggest possible epigenetic, transcriptionally driven remodeling under antiestrogen pressures.

Nearly all recurrences are more similar transcriptionally to their matched primaries than to other, long-term estrogen-deprived tumors—reinforcing the notion that advanced cancers generally retain their core transcriptional programming, even after nearly a decade of dormancy [26–29]. Furthermore, amplifications and deletions of recurrences are markedly similar to primaries, supporting recent evidence from breast cancer single-cell sequencing that structural variation is likely an early event and many CNAs, even in metachronous therapy-resistant tumors, may be shared by the majority of subclones [57]. An important exception to this conservation was ERLR_03_R1, a recurrence with a completely unique transcriptional and copy number profile than its matched primary. Evidence has emerged of so-called collision tumors, whereby two synchronous, distinct cancers can merge anatomically and only under the selective pressures of therapy or through deep sequencing, their individuality can be unmasked [23, 58]. Indeed, this “recurrence” switched to ER-negative/HER2-positive from ER-positive/HER2-negative clinically and thus could represent a different cancer than the primary—although the level of shared SNVs suggests some degree of clonal relatedness.

Limited shared, non-silent SNVs were discovered in these specimens, with *AKAP9* and *KMT2C* being the only two genes that harbored recurrence-enriched mutations in greater than one case. These mutations are not in a conserved functional domain nor in a hotspot location, making it difficult to assess their pathogenic roles. *AKAP9* and *KMT2C* also encode relatively large gene products (3911 and 4911 amino acids, respectively) which may increase the likelihood of obtaining a passenger mutation by chance. Nevertheless, *KMT2C* and other lysine methyltransferases have been implicated in breast cancer pathology, argued as potential drivers in large-scale sequencing studies of primary tumors and *KMT2C* mutations specifically may confer hormone therapy resistance in breast cancer models [59–61]. Case ERLR_20 harbored an enriched nonsense mutation in *ARID1A*. *ARID1A* alterations are associated with more unfavorable tumor features in breast cancer and have recently been shown to determine luminal identity and therapy response in ER-positive tumors—consistent with the more basal-like transcriptional features we observe with *ESR1*-depleted recurrences [62–65]. A single recurrent cancer (ERLR_01_R1) showed enrichment of three somatic hotspot *PIK3CA* mutations (E542K, Q546K, E726K), suggesting strong MAPK signaling selection within that particular tumor and coincident with recent reports of multiple mutations occurring in individual cancer genes in advanced cancers [66]. SNVs within genes that act as corepressors and coactivators, some with direct influences on estrogen receptor-mediated transcription, were found to be enriched in recurrences—such as *NCOA1*, *NCOR2*, *FRYL*, and *CREBBP*—along with transcription factors including *PAX5*, *FOXO1*, and *TP53*. Notably, we did not observe any *ESR1* mutations unlike other studies on locoregional recurrences [67]—likely due to our small sample size. Interestingly, this study reported lower frequency of *ESR1* mutations in locoregional recurrences versus advanced metastases at an AF > 1% and recent data has emerged regarding a pro-metastatic phenotype of *ESR1* variants [68]—suggesting locoregional recurrences may have a lower frequency of *ESR1* variants versus distant disease. We also observed a positive correlation between the frequency of acquired, non-silent SNVs and disease-free survival—validating the concept that surviving cancer cells after initial therapy acquire potentially pathogenic mutations as they lay dormant and undetectable over time.

Given the heterogeneity of clinical specimens makes it difficult to rely on typically used differential expression workflows—since resistant mechanisms of individual tumors may be distinct—we undertook an analysis of patient-specific outlier expression gains and losses to identify more extreme transcriptional reprogramming events within individual cases that may be driving estrogen independence. Surprisingly, unlike SNVs, recurrent outlier transcriptional gains and losses were quite common*.* Particularly compelling outlier events included recurrent gains within shared pathway members, such as near mutually exclusive upregulations of *NTRK3* (*n* = 5 [42%]) and *NTRK2* (*n* = 4 [33%]). Notably, activation of *NTRK’s* mediates downstream signaling pathways typically associated with breast carcinomas, including PI3K and MAPK, and small molecule inhibitors of this family are showing promising results in recent solid tumor trials [69]. Other notable pathway member changes included loss of Wnt antagonists *SFRP2* (*n* = 3 [25%]) and *SFRP4* (*n* = 4 [33%]). *SFRP2* is hypermethylated and silenced in a subset of breast cancers [70] and experiments in model systems have shown cross-talk between ER and Wnt signaling that may mediate endocrine therapy resistance [71, 72]. Other recurrent gains included *FGFR4* (*n* = 4 [33%]), *TERT* (*n* = 3 [25%]), and *CCNE1* (*n* = 3 [25%])*—*particularly relevant given the recent success of CDK inhibitors in hormone-positive disease and the burgeoning use of *FGFR* inhibitors against solid malignancies as we and others have reported [31, 73].

The most recurrent outlier expression loss was *ESR1*, which was diminished in 42% of long-term estrogen-deprived local recurrences. Interestingly, the loss of *ESR1* for the majority of cases was not associated with a dramatic change in the tumors’ transcriptional profile. To further explore this counterintuitive result, given *ESR1* is a master regulator of transcription and a driver of luminal breast cancers, we identified genes that were consistently altered in *ESR1-*depleted recurrences. The most substantial gains in *ESR1-*depleted tumors are genes generally expressed in basal breast cancers—such as *NDRG1*, *DKK1*, *KIT*, *KLK7*, *PROM1*, and *COL9A3*—and genes significantly lost in the *ESR1-*depleted subset are generally downregulated in basal cancers—*EVLOVL2*, *BCL2*, *IGF1R*, *MYB*, *RABEP*, and *ATP8A2* (MsigDB: SMID_BREAST_CANCER_BASAL_DN/UP gene lists) [74]. These results reveal a common, novel, and distinct *ESR1*-depleted subtype of advanced breast cancers that acquire basal-like transcriptional reprogramming. Prior studies have hinted that luminal B tumors, which are known to portend worse outcomes, generally have lower expression of *ESR1* and endocrine-resistant tumors have been shown to have decreased *ESR1* expression relative to matched primary tumors [75]. The mechanisms driving this loss as well as the *ESR1*-independent maintenance of a luminal cell-state with basal-like characteristics will be essential to unravel. Interestingly, prior studies have shown that intrinsic molecular subtypes of breast cancers generally remain consistent in recurrent or metastatic tumors, yet here we see a more nuanced gain of basal-like features in luminal tumors [76–78].

The greatest fold-change difference in *ESR1-*depleted recurrences was the upregulation of *PROM1*. *PROM1* is a marker for tumor-initiating cancer stem cells and plays a key role in determining ER-positive luminal cell fate during differentiation from multipotent stem cells [56], suggesting long-term endocrine-deprived breast cancer cells may enrich themselves with stem-like progenitors to achieve estrogen independence. Indeed, *PROM1* has been shown to mediate endocrine therapy resistance in breast cancer models through IL6/Notch3 signaling [79, 80]. Here, we show that a large portion of long-term endocrine-resistant breast cancers may be exploiting this transcriptional reprogramming. Finally, *NDRG1*, also significantly upregulated in *ESR1-*depleted recurrences and generally expressed in basal cancers, showed differential expression in three distinct LTED cell lines. *NDRG1* is a suspected metastasis suppressor gene. Counterintuitively, we see upregulation of this gene in resistant disease and show increased expression confers worse survival outcomes in ER-positive primary tumors [81]. Further functional studies assessing the mechanistic and biological consequences of these transcriptional reprogramming events both in locoregional and metastatic disease will be essential.

A pertinent point these results raise is the benefit of integrating longitudinal, targeted RNA sequencing to inform resistance mechanisms and therapeutic targets in breast cancers. In this study, we find limited DNA-level enrichments yet highly recurrent, acquired transcriptional remodeling events from primary to advanced cancers, including a few of which that are immediately targetable such as *NTRKs*, *FGFR4*, and *CCNE1—*although this study was limited by the small number of patient-matched cases and targeted panel of genes. Nonetheless, this work challenges our lack of emphasis on RNA-level changes, particularly those that can be elucidated from longitudinal biopsies, in clinical profiling of tumors and future work should be geared towards deciphering which of these bypass transcriptional programs may be druggable.

## Conclusions

Collectively, these results begin to unravel the complex adaptations that breast cancer populations undergo when under the selection of long-term estrogen-depleting therapies long term. We identify acquired DNA-level mechanisms of resistance, such as mutations in *ARID1A*, other transcriptional regulators, and multiple mutation selection within *PIK3CA*—but more importantly, uncover the most recurrent genomic adaptations taking place appear to be at the transcriptional level. These include targetable outlier gains and modifications in *NTRKs* as well as a distinct population of *ESR1-*depleted recurrences that enrich themselves with genes generally expressed in basal breast cancers—such as *PROM1* and *NDRG1*. Preclinical, mechanistic investigations into these temporally altered genes are warranted given they may uncover novel and targetable mechanisms of endocrine therapy resistance in advanced breast cancers.

## Supplementary Information

**Additional file 1 **Title of Data: **Supplementary Figures**, Description of Data: Figures supporting manuscript labeled Supplementary Figure 1 through 9.

**Additional file 2 **Title of Data: **Data Supplement**, Description of Data: Supplemental numerical data supporting manuscript labeled in excel tabs as S1-S9.

## Data Availability

The datasets analyzed and generated for this study are available from the corresponding author on request pending institutional data use authorization agreements.

## References

[1] Early Breast Cancer Trialists’ Collaborative Group (EBCTCG) (2011). Relevance of breast cancer hormone receptors and other factors to the efficacy of adjuvant tamoxifen: patient-level meta-analysis of randomised trials. Lancet.

[2] Davies C (2013). Long-term effects of continuing adjuvant tamoxifen to 10 years versus stopping at 5 years after diagnosis of oestrogen receptor-positive breast cancer: ATLAS, a randomised trial. Lancet.

[3] Goss PE (2016). Extending aromatase-inhibitor adjuvant therapy to 10 years. N Engl J Med.

[4] Jacobson JA (1995). Ten-year results of a comparison of conservation with mastectomy in the treatment of stage I and II breast cancer. N Engl J Med.

[5] Robinson DR (2013). Activating ESR1 mutations in hormone-resistant metastatic breast cancer. Nat Genet.

[6] Toy W (2013). ESR1 ligand-binding domain mutations in hormone-resistant breast cancer. Nat Genet.

[7] Jeselsohn R, Buchwalter G, De Angelis C, Brown M, Schiff R (2015). ESR1 mutations—a mechanism for acquired endocrine resistance in breast cancer. Nat Rev Clin Oncol.

[8] Kuukasjärvi T, Kononen J, Helin H, Holli K, Isola J (1996). Loss of estrogen receptor in recurrent breast cancer is associated with poor response to endocrine therapy. J Clin Oncol.

[9] Oh AS (2001). Hyperactivation of MAPK induces loss of ERalpha expression in breast cancer cells. Mol Endocrinol.

[10] Creighton CJ (2006). Activation of mitogen-activated protein kinase in estrogen receptor alpha-positive breast cancer cells in vitro induces an in vivo molecular phenotype of estrogen receptor alpha-negative human breast tumors. Cancer Res.

[11] Shou J (2004). Mechanisms of tamoxifen resistance: increased estrogen receptor-HER2/neu cross-talk in ER/HER2-positive breast cancer. J Natl Cancer Inst.

[12] Turner N (2010). FGFR1 amplification drives endocrine therapy resistance and is a therapeutic target in breast cancer. Cancer Res.

[13] Basudan A (2019). Frequent ESR1 and CDK pathway copy-number alterations in metastatic breast cancer. Mol Cancer Res.

[14] Hartmaier RJ (2018). Recurrent hyperactive ESR1 fusion proteins in endocrine therapy-resistant breast cancer. Ann Oncol.

[15] Gundem G (2015). The evolutionary history of lethal metastatic prostate cancer. Nature.

[16] Hugo W (2015). Non-genomic and immune evolution of melanoma acquiring MAPKi resistance. Cell.

[17] Nik-Zainal S (2012). The life history of 21 breast cancers. Cell.

[18] Hanker AB (2017). An acquired HER2T798I gatekeeper mutation induces resistance to neratinib in a patient with HER2 mutant-driven breast cancer. Cancer Discov.

[19] Miller WR (2007). Changes in breast cancer transcriptional profiles after treatment with the aromatase inhibitor, letrozole. Pharmacogenet Genomics.

[20] Mackay A (2007). Molecular response to aromatase inhibitor treatment in primary breast cancer. Breast Cancer Res.

[21] Gutierrez MC (2005). Molecular changes in tamoxifen-resistant breast cancer: relationship between estrogen receptor, HER-2, and p38 mitogen-activated protein kinase. J Clin Oncol.

[22] Varešlija D (2016). Adaptation to AI therapy in breast cancer can induce dynamic alterations in ER activity resulting in estrogen-independent metastatic tumors. Clin Cancer Res.

[23] Miller CA (2016). Aromatase inhibition remodels the clonal architecture of estrogen-receptor-positive breast cancers. Nat Commun.

[24] Razavi P (2018). The genomic landscape of endocrine-resistant advanced breast cancers. Cancer Cell.

[25] Nayar U (2019). Acquired HER2 mutations in ER+ metastatic breast cancer confer resistance to estrogen receptor-directed therapies. Nat Genet.

[26] Pearson A (2020). Inactivating NF1 mutations are enriched in advanced breast cancer and contribute to endocrine therapy resistance. Clin Cancer Res.

[27] Zheng Z-Y (2020). Neurofibromin is an estrogen receptor-α transcriptional co-repressor in breast cancer. Cancer Cell.

[28] Robinson DR (2017). Integrative clinical genomics of metastatic cancer. Nature.

[29] Varešlija D (2019). Transcriptome characterization of matched primary breast and brain metastatic tumors to detect novel actionable targets. J Natl Cancer Inst.

[30] Priedigkeit N, et al. Exome-capture RNA sequencing of decade-old breast cancers and matched decalcified bone metastases. JCI Insight. 2017;2(17):e95703. 10.1172/jci.insight.95703 PMC5621874 28878133

[31] Levine KM (2019). FGFR4 overexpression and hotspot mutations in metastatic ER+ breast cancer are enriched in the lobular subtype. NPJ Breast Cancer.

[32] Patro R, Duggal G, Love MI, Irizarry RA, Kingsford C (2017). Salmon provides fast and bias-aware quantification of transcript expression. Nat Methods.

[33] Soneson C, Love MI, Robinson MD (2016). Differential analyses for RNA-seq: transcript-level estimates improve gene-level inferences. [version 2; peer review: 2 approved]. F1000Res.

[34] Robinson MD, McCarthy DJ, Smyth GK (2010). edgeR: a Bioconductor package for differential expression analysis of digital gene expression data. Bioinformatics.

[35] Robinson MD, Oshlack A (2010). A scaling normalization method for differential expression analysis of RNA-seq data. Genome Biol.

[36] Li H, Durbin R (2009). Fast and accurate short read alignment with Burrows-Wheeler transform. Bioinformatics (Oxford).

[37] Li H (2009). The sequence alignment/map format and SAMtools. Bioinformatics (Oxford).

[38] McKenna A (2010). The Genome Analysis Toolkit: a MapReduce framework for analyzing next-generation DNA sequencing data. Genome Res.

[39] Koboldt DC (2012). VarScan 2: somatic mutation and copy number alteration discovery in cancer by exome sequencing. Genome Res.

[40] Obenchain V (2014). VariantAnnotation: a Bioconductor package for exploration and annotation of genetic variants. Bioinformatics.

[41] Wong SQ (2014). Sequence artefacts in a prospective series of formalin-fixed tumours tested for mutations in hotspot regions by massively parallel sequencing. BMC Med Genet.

[42] Wang K, Li M, Hakonarson H (2010). ANNOVAR: functional annotation of genetic variants from high-throughput sequencing data. Nucleic Acids Res.

[43] Lek M (2016). Analysis of protein-coding genetic variation in 60,706 humans. Nature.

[44] Gu Z, Eils R, Schlesner M. Complex heatmaps reveal patterns and correlations in multidimensional genomic data. Bioinformatics. 2016;32(18):2847-9. 10.1093/bioinformatics/btw313 27207943

[45] Robinson JT (2011). Integrative genomics viewer. Nat Biotechnol.

[46] Talevich E, Shain AH, Botton T, Bastian BC (2016). CNVkit: genome-wide copy number detection and visualization from targeted DNA sequencing. Plos Comput Biol.

[47] Olshen AB, Venkatraman ES, Lucito R, Wigler M (2004). Circular binary segmentation for the analysis of array-based DNA copy number data. Biostatistics.

[48] Law CW, Chen Y, Shi W, Smyth GK (2014). voom: precision weights unlock linear model analysis tools for RNA-seq read counts. Genome Biol.

[49] Ritchie ME (2015). limma powers differential expression analyses for RNA-sequencing and microarray studies. Nucleic Acids Res.

[50] Curtis C (2012). The genomic and transcriptomic architecture of 2,000 breast tumours reveals novel subgroups. Nature.

[51] Simigdala N (2016). Cholesterol biosynthesis pathway as a novel mechanism of resistance to estrogen deprivation in estrogen receptor-positive breast cancer. Breast Cancer Res.

[52] Bland JM, Altman DG (2004). The logrank test. BMJ.

[53] Kassambara A, Kosinski M. survminer: drawing survival curves using ggplot2. R package version 0.4.8. https://CRAN.R-project.org/package=survminer.

[54] Forbes SA (2015). COSMIC: exploring the world’s knowledge of somatic mutations in human cancer. Nucleic Acids Res.

[55] Du T (2018). Key regulators of lipid metabolism drive endocrine resistance in invasive lobular breast cancer. Breast Cancer Res.

[56] Wang C, Christin JR, Oktay MH, Guo W (2017). Lineage-biased stem cells maintain estrogen-receptor-positive and -negative mouse mammary luminal lineages. Cell Rep.

[57] Gao R (2016). Punctuated copy number evolution and clonal stasis in triple-negative breast cancer. Nat Genet.

[58] Wahner-Roedler DL, Reynolds CA, Boughey JC (2011). Collision tumors with synchronous presentation of breast carcinoma and lymphoproliferative disorders in the axillary nodes of patients with newly diagnosed breast cancer: a case series. Clin Breast Cancer.

[59] Liu L, Kimball S, Liu H, Holowatyj A, Yang Z-Q (2015). Genetic alterations of histone lysine methyltransferases and their significance in breast cancer. Oncotarget.

[60] Pereira B (2016). The somatic mutation profiles of 2,433 breast cancers refines their genomic and transcriptomic landscapes. Nat Commun.

[61] Manso L (2016). Analysis of paired primary-metastatic hormone-receptor positive breast tumors (HRPBC) uncovers potential novel drivers of hormonal resistance. PLoS One.

[62] Jones S (2010). Frequent mutations of chromatin remodeling gene ARID1A in ovarian clear cell carcinoma. Science.

[63] Guan B, Wang T-L, Shih I-M (2011). ARID1A, a factor that promotes formation of SWI/SNF-mediated chromatin remodeling, is a tumor suppressor in gynecologic cancers. Cancer Res.

[64] Zhang X (2012). Frequent low expression of chromatin remodeling gene ARID1A in breast cancer and its clinical significance. Cancer Epidemiol.

[65] Xu G (2020). ARID1A determines luminal identity and therapeutic response in estrogen-receptor-positive breast cancer. Nat Genet.

[66] Saito Y (2020). Landscape and function of multiple mutations within individual oncogenes. Nature.

[67] Zundelevich A (2020). ESR1 mutations are frequent in newly diagnosed metastatic and loco-regional recurrence of endocrine-treated breast cancer and carry worse prognosis. Breast Cancer Res.

[68] Jeselsohn R (2018). Allele-specific chromatin recruitment and therapeutic vulnerabilities of ESR1 activating mutations. Cancer Cell.

[69] Drilon A (2018). Efficacy of larotrectinib in TRK fusion-positive cancers in adults and children. N Engl J Med.

[70] Veeck J (2008). Promoter hypermethylation of the SFRP2 gene is a high-frequent alteration and tumor-specific epigenetic marker in human breast cancer. Mol Cancer.

[71] Loh YN (2013). The Wnt signalling pathway is upregulated in an in vitro model of acquired tamoxifen resistant breast cancer. BMC Cancer.

[72] Sikora MJ (2016). WNT4 mediates estrogen receptor signaling and endocrine resistance in invasive lobular carcinoma cell lines. Breast Cancer Res.

[73] Touat M, Ileana E, Postel-Vinay S, André F, Soria J-C (2015). Targeting FGFR signaling in cancer. Clin Cancer Res.

[74] Subramanian A (2005). Gene set enrichment analysis: a knowledge-based approach for interpreting genome-wide expression profiles. Proc Natl Acad Sci U S A.

[75] Johnston SR (1995). Changes in estrogen receptor, progesterone receptor, and pS2 expression in tamoxifen-resistant human breast cancer. Cancer Res.

[76] Cejalvo JM (2017). Intrinsic subtypes and gene expression profiles in primary and metastatic breast cancer. Cancer Res.

[77] Siegel MB (2018). Integrated RNA and DNA sequencing reveals early drivers of metastatic breast cancer. J Clin Invest.

[78] Priedigkeit N (2017). Intrinsic subtype switching and acquired ERBB2/HER2 amplifications and mutations in breast cancer brain metastases. JAMA Oncol.

[79] Sansone P (2016). Self-renewal of CD133(hi) cells by IL6/Notch3 signalling regulates endocrine resistance in metastatic breast cancer. Nat Commun.

[80] Sansone P (2017). Evolution of cancer stem-like cells in endocrine-resistant metastatic breast cancers is mediated by stromal microvesicles. Cancer Res.

[81] Guan RJ (2000). Drg-1 as a differentiation-related, putative metastatic suppressor gene in human colon cancer. Cancer Res.

