# Supplementary material for: Acquired mutations and transcriptional remodeling in long-term estrogen-deprived locoregional breast cancer recurrences
Source: Breast Cancer Res. 2021 Jan 6;23:1. doi: 10.1186/s13058-020-01379-3 (PMC7788918; doi:10.1186/s13058-020-01379-3)
Supplement: Supplementary file 1 — Additional file 1 Title of Data: Supplementary Figures, Description of Data: Figures supporting manuscript labeled Supplementary Figure 1 through 9. [file 13058_2020_1379_MOESM1_ESM.pdf]

SUPPLEMENTARY FIGURES

Acquired mutations and transcriptional remodeling in long-term estrogen-deprived locoregional breast cancer recurrences

Nolan Priedigkeit, Kai Ding, William Horne, Jay K. Kolls, Tian Du, Peter C. Lucas, Jens-Uwe Blohmer, Carsten Denkert, Anna Machleidt, Barbara Ingold Heppner, Steffi Oesterreich, Adrian V. Lee

Supplementary Figure 1. DNA-seq target interval coverages

Supplementary Figure 2. Copy number call distribution

Supplementary Figure 3. tumorMatch: Proportion of shared variants (POSV) between samples in patient-matched cohort

Supplementary Figure 4. Local recurrence CNA Correlation Matrix

Supplementary Figure 5. Allele frequency of SNVs called from RNA-seq vs DNA-seq

Supplementary Figure 6. Overlap of differentially expressed genes between local recurrences and ER+ LTED lines

Supplementary Figure 7. *KLK7* and *PROM1* basal breast carcinoma expression and E2 regulation.

Supplementary Figure 8. NDRG1 expression in PAM50 subtypes and survival influence in ER-positive breast cancer.

Supplementary Figure 9: Top five up and downregulated ESR1-depleted gene expression changes in ESR1 non-depleted cases.

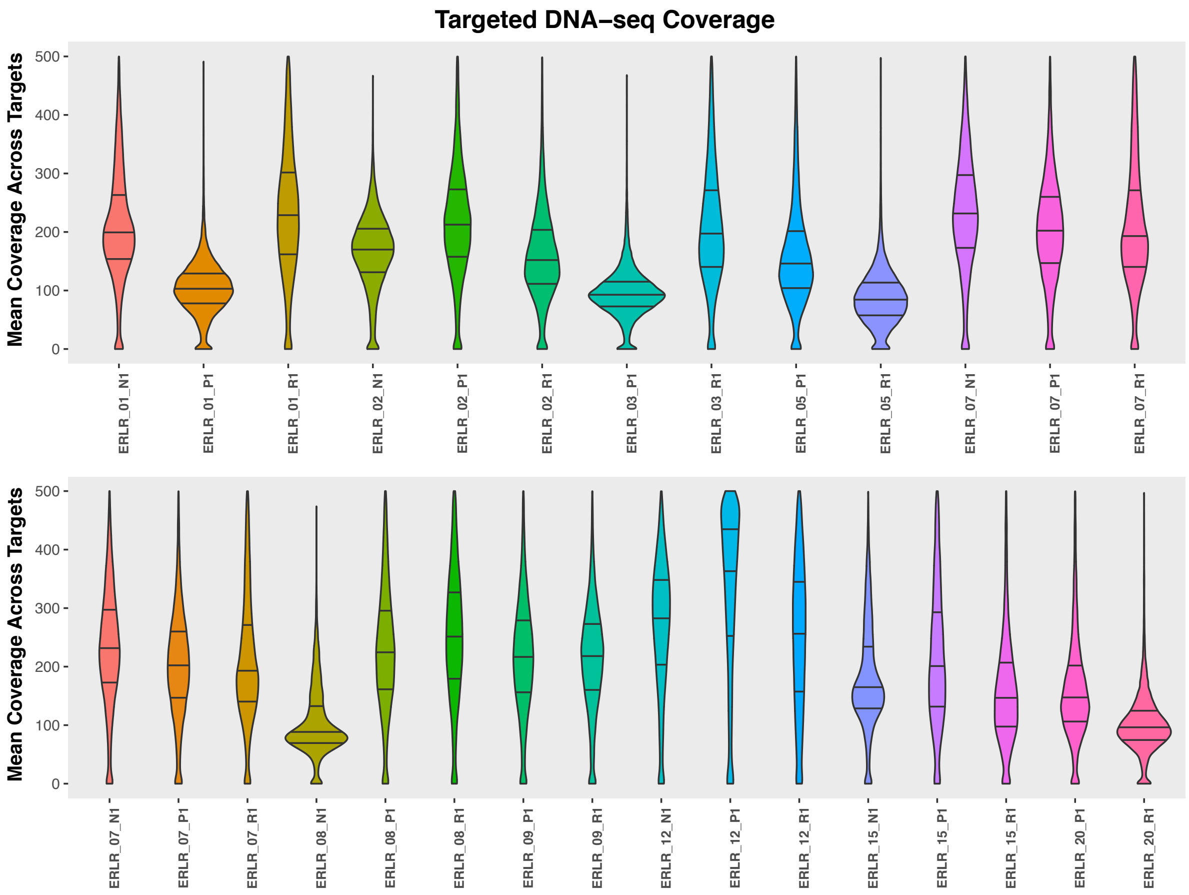

Supplementary Figure 1: ***DNA-seq target interval coverages.*** Violin plots showing the distribution of mean DNA-sequencing coverage across all targeted intervals. 25th, 50th and 75th percentiles are indicated with horizontal black lines. To better visualize distributions, y-axis limit was set at 500.

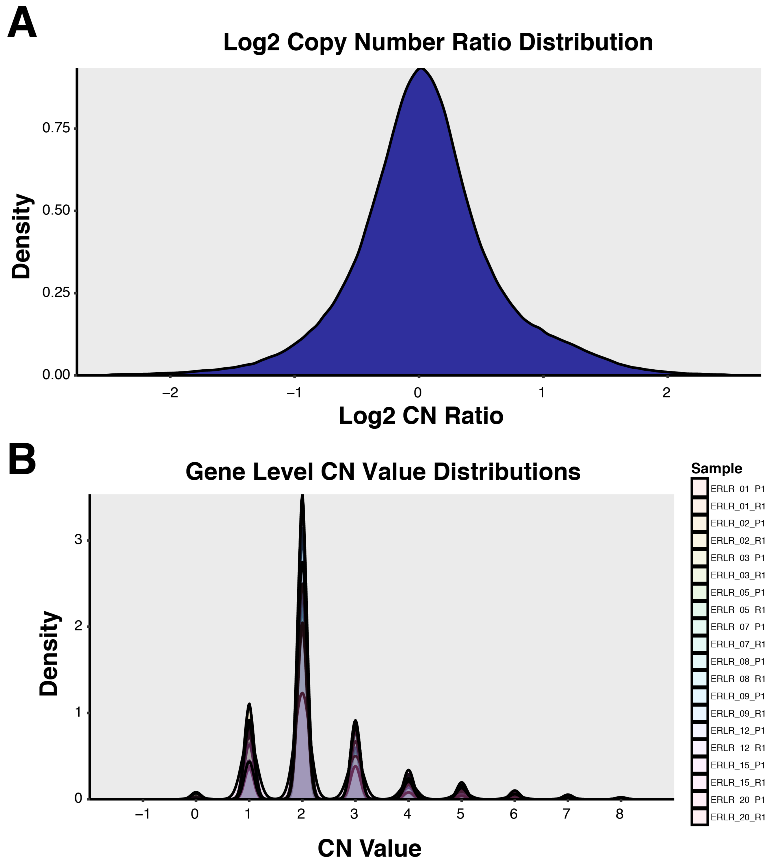

Supplementary Figure 2: ***Copy number call distribution.*** **(A)** Log2 copy number ratio distribution, derived from *CNVkit*, for all samples in cohort. **(B)** Distribution of discrete, gene-level copy number calls with gene-level values representing the mean of discrete calls across all probed regions covering the gene.

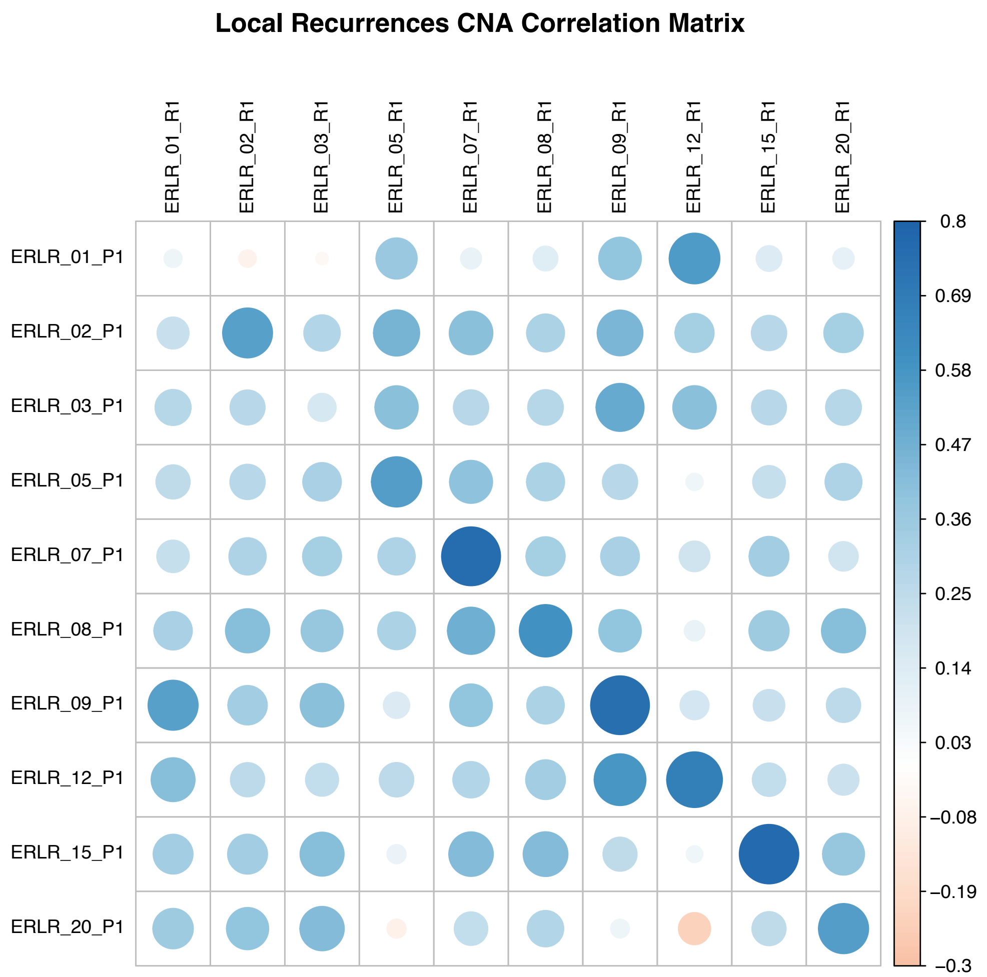

Supplementary Figure 3: ***CNA Correlation Matrix.*** Correlation matrix of pearson R values from log2CN values between all samples.

tumorMatch **in local recurrence cohort**

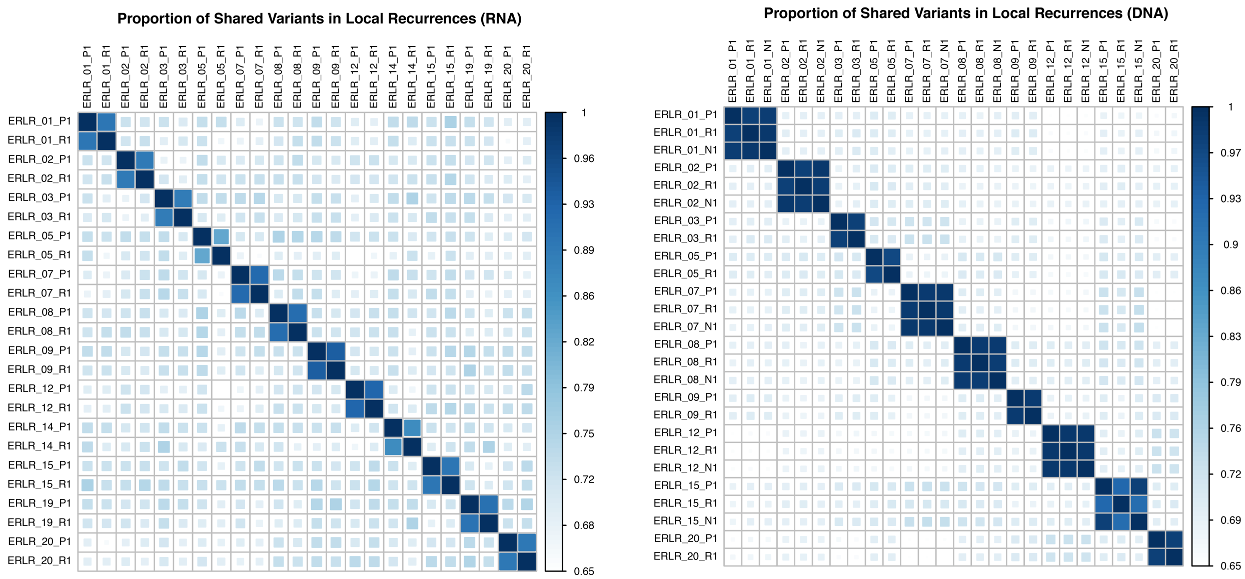

<u>Supplementary Figure 4:</u> tumorMatch proportion of shared variants (POSV) between samples in patient-matched cohort. *tumorMatch* plots for both RNA- (left) and DNA-sequencing, showing all paired specimens, including trios that include normal, are patient-matched.

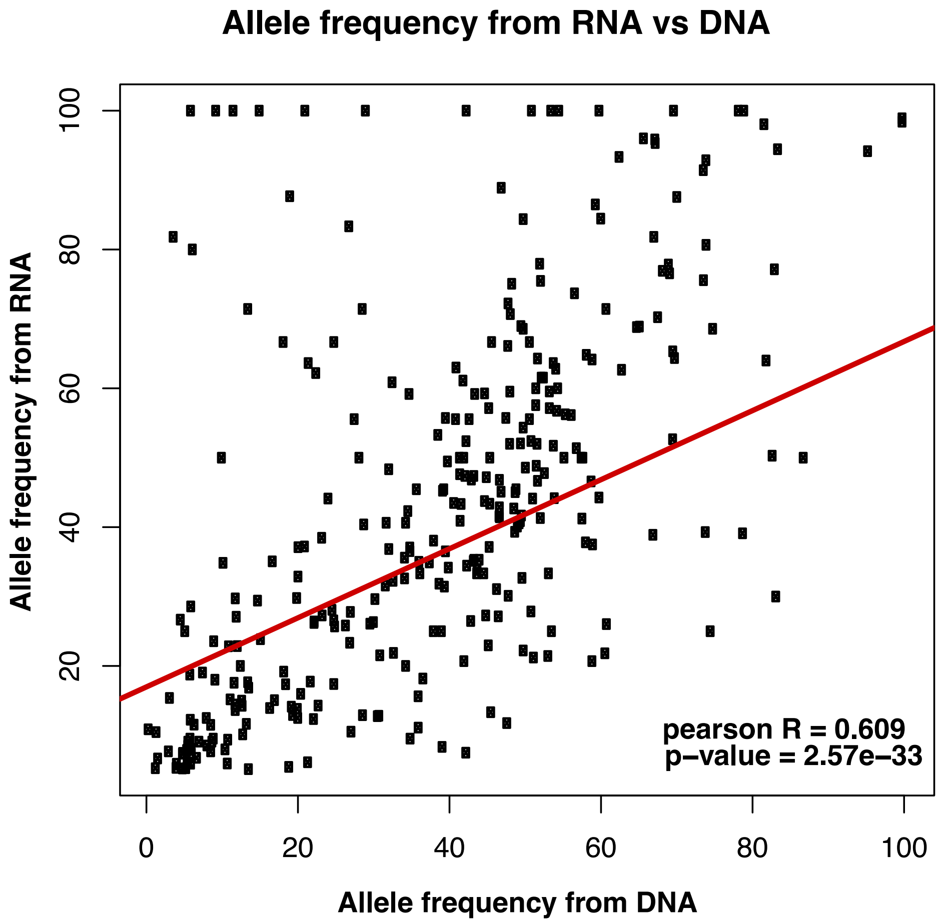

Supplementary Figure 5: **Allele frequency of SNVs called from RNA-seq vs DNA-seq.**

Allele frequency calls of 633 total somatic, nonsynonymous nucleotide variants from RNA-seq and DNA-seq (pearson R = 0.609).

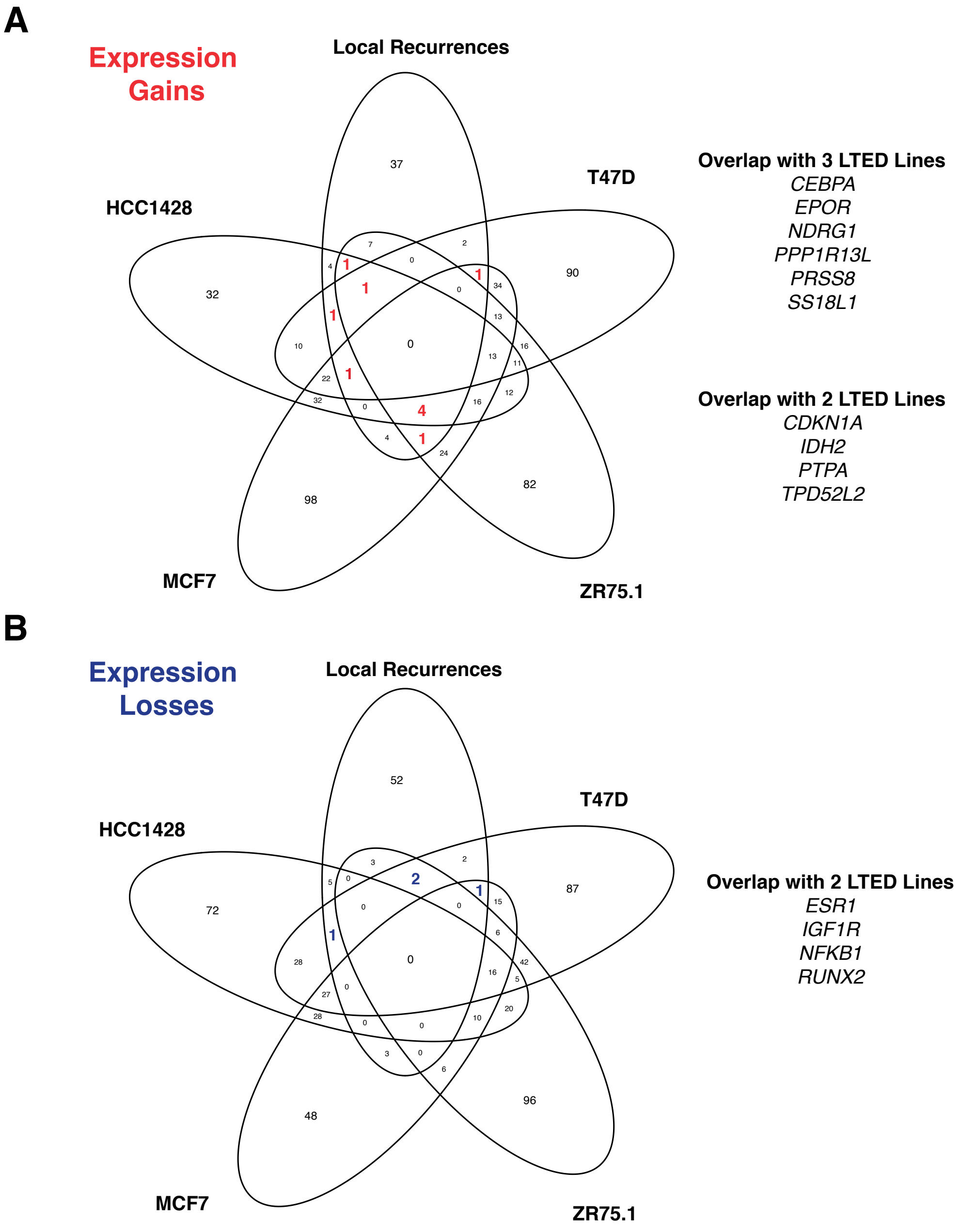

Supplementary Figure 6: **Overlap of differentially expressed genes between local recurrences and ER+ LTED lines. (A)** Genes significantly upregulated in both local recurrences vs. primaries and LTED vs. parental lines. **(B)** Genes significantly downregulated in both local recurrences vs. primaries and LTED vs. parental lines.

A

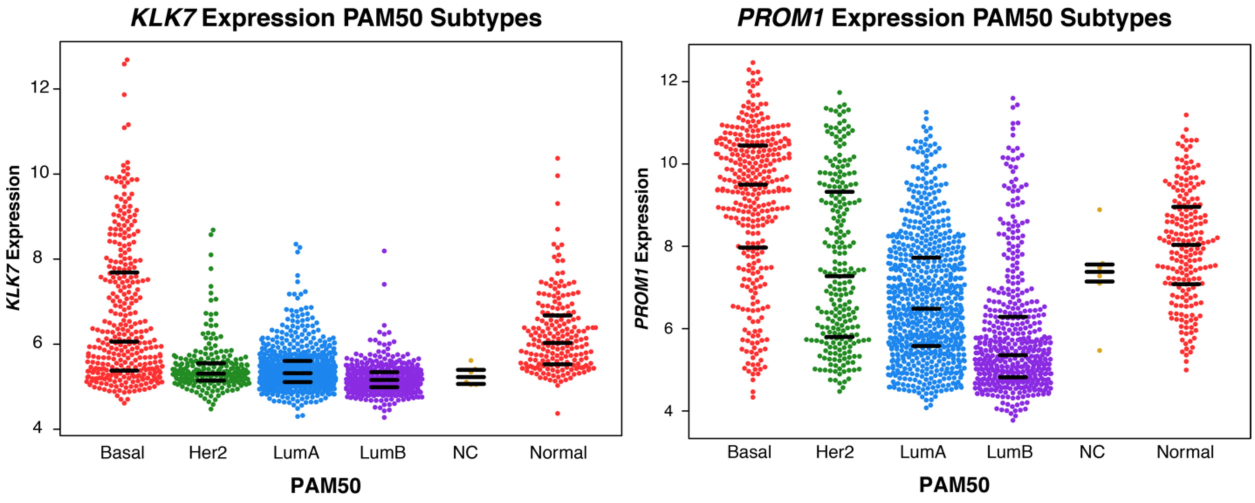

B

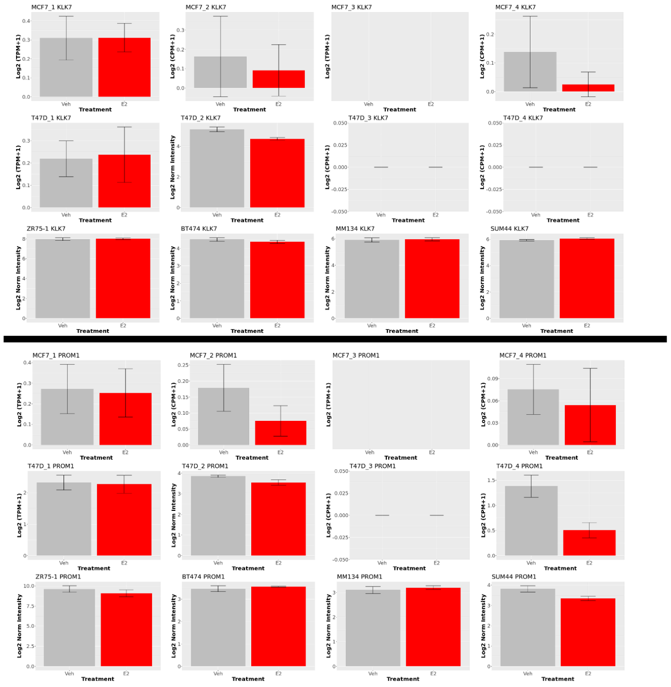

Plot	Condition	Data.Type	GEO
MCF7_1	1nM, 24h	RNA-seq	GSE89888
MCF7_2	10 nM, 24h	RNA-seq	GSE51403
MCF7_3	10 nM, 24h	RNA-seq	GSE78286
MCF7_4	1 nM, 24h	RNA-seq	GSE94493
T47D_1	1nM, 24h	RNA-seq	GSE89888
T47D_2	1 nM, 24h	Microarray	GSE3834
T47D_3	10 nM, 24h	RNA-seq	GSE94493
T47D_4	1 nM, 24h	RNA-seq	GSE108304
ZR75-1	10 nM, 16h	Microarray	GSE61368
BT474	1 nM, 24h	Microarray	GSE3834
MM134	1 nM, 24h	Microarray	GSE50695
SUM44	1 nM, 24h	Microarray	GSE50695

<u>Supplementary Figure 7:</u> ***KLK7*** **and** ***PROM1*** **basal breast carcinoma expression and E2 regulation. (A)** Normalized microarray expression values (METABRIC) of *KLK7* and *PROM1*, segregated by PAM50 subtypes. Horizontal black bars indicate 25th, 50th and 75th percentile values. **(B)** Relative expression of *KLK7* and *PROM1* with E2 induction in various conditions and breast cancer cell lines.

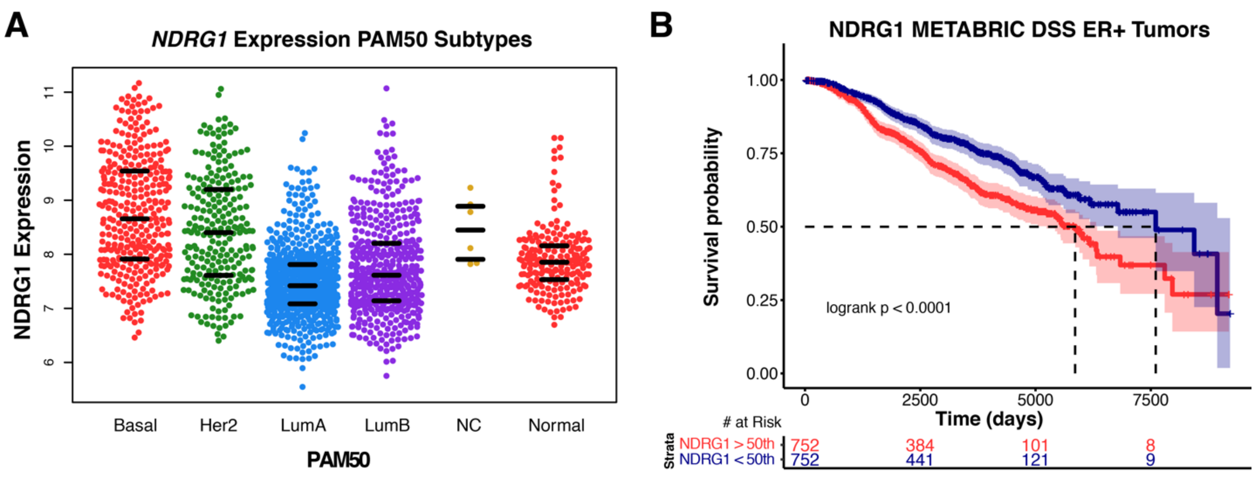

Supplementary Figure 8: **NDRG1 expression in PAM50 subtypes and survival influence in ER-positive breast cancer. (A)** *NDRG1* expression in PAM50 subtypes. **(B)** Disease-specific survival in METABRIC of patients with ER-positive primary tumors that express *NDRG1* highly (>50th percentile, red) or lowly (<50th percentile, blue).

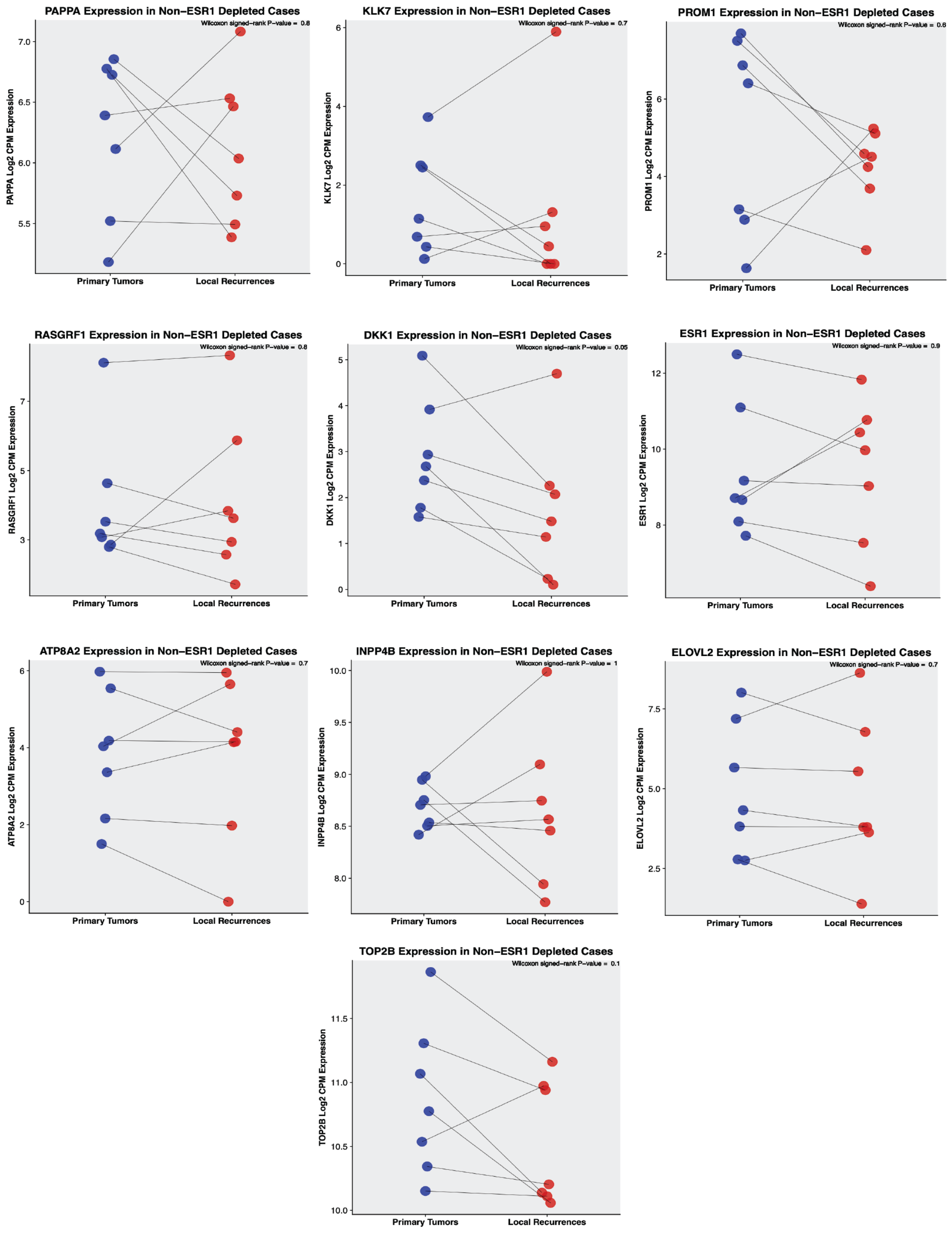

Supplementary Figure 9: **Top five up and downregulated ESR1-depleted gene expression changes in ESR1 non-depleted cases.** Plots of log2CPM expression values of primary (blue) and recurrent (red) tumors from non-ESR1 depleted cases and associated Wilcoxon signed-rank P-values.
